# Commentary in reply to a publication on *Plasmodium falciparum* pre-erythrocytic stage vaccine development

**DOI:** 10.1186/s12936-020-03334-1

**Published:** 2020-07-20

**Authors:** François P. Roman, Margherita Coccia, Lode Schuerman

**Affiliations:** grid.425090.a GSK, Avenue Fleming 20, 1300 Wavre, Belgium

**Keywords:** Malaria vaccine, Clinical studies, RTS,S/AS01

## Abstract

We have read the publication of Molina-Franky and colleagues on *Plasmodium falciparum* pre-erythrocytic stage vaccine development (Malaria Journal, 2020;19:56). The commentary revises some of their statements on the RTS,S/AS01 vaccine that are considered either imprecise or incorrect.

## Main text

While we agree that efforts to find and introduce new candidate pre-erythrocytic malaria vaccines are warranted, we disagree with the statement of Molina-Franky and colleagues [1] that none of these approaches has led to promising results regarding an effective control of the disease. It is indeed our opinion that the results available today for RTS,S/AS01, and based on at least 28 clinical Phase I, II and III studies [2] (and not five studies as suggested in Table 1 of the paper) do indicate the potential of this vaccine to provide substantial public health benefit. This is well supported by the European Medicine Agency (EMA) (as reflected in their positive scientific opinion [3]), the World Health Organization (WHO) (as reflected in the WHO position paper on this vaccine [4]) and the national regulatory authorities in Ghana, Kenya and Malawi [5].

We would also like to address some of the specific points raised by the paper. The authors state that “A tetrapeptide from the CSP NANP tandem repeat region (R) and the C-terminal region containing T cell (T) epitopes (exclusive for the NF54 strain) are fused to the hepatitis B surface (S) antigen”. However, each RTS molecule includes 19 copies of the tetrapeptide repeat motif (NANP). In addition, the fact that the C-terminal region of the CSP protein included in the vaccine is derived from the NF54 parasite strain does not mean that it is exclusive for that strain [6–8].

With reference to the WHO position paper on Malaria vaccine [4], the authors state that the EMA issued a “cautious scientific opinion regarding its quality”. However, neither the WHO position paper, nor EMA, qualified the positive scientific opinion as cautious, and no specific caution was expressed on the quality of the RTS,S/AS01 vaccine in particular.

The authors also address a series of other concerns relating to the profile of RTS,S/AS01 or to some of its components, such as the genetic variability of the selected CSP region, high parasitaemia levels in “individuals considered protected”, presumed pro-apoptotic signals induced by some RTS,S components, or a lack of mechanistic understanding of the AS01 adjuvant system used in the vaccine.

Regarding CSP variability, we know that in the large phase III trial where efficacy was demonstrated against malaria, fewer than 10% of the parasites matched the CSP protein alleles used in the vaccine [9]. These reassuring data clearly mitigate the concern that CSP variability may neutralize vaccine efficacy.

High parasitaemia levels (i.e. > 5000 parasites/μl or 0.1% parasitaemia) are not unexpected after RTS,S vaccination, since the objective of the vaccination is not to prevent infection but to reduce the risk of clinical episodes of malaria. During RTS,S vaccine development the > 5000 parasites/μl threshold was used to define clinical cases of malaria to evaluate vaccine efficacy, but not as a direct efficacy surrogate of the vaccine [10–14]. A child with a parasitaemia of 5000 parasites/µl or more was not considered to be protected, but on the contrary to experience a malaria episode.

It is unclear why the authors attribute pro-apoptotic signals to RTS,S components, based on the references provided in the manuscript [15, 16]. From our own review on the two cited papers, such pro-apoptotic signals are described as potential malaria disease-related mechanisms, or as B-cell based mechanisms in the context of influenza vaccination, but not as RTS,S/AS01-related mechanisms. Importantly, the actual clinical implications of this statement are unclear.

Finally, albeit the mechanism of action of AS01 is indeed complex, significant published work exists now that clarify many of its aspects [17, 18], including the mechanisms of QS21 when formulated in liposomes [19, 20].

## Data Availability

The authors declare that all data supporting the findings of this study are available within the article and its additional files.
